# Health Effects of Long-Term Exposure to Insecticide-Treated Mosquito Nets in the Control of Malaria in Endemic Regions, Revised

**DOI:** 10.1100/tsw.2006.272

**Published:** 2006-12-15

**Authors:** Ebere C. Anyanwu, John E. Ehiri, Ijeoma Kanu, Joav Merrick

**Affiliations:** ^1^ Cahers Research C/o 41 MCH, 190 College Road, Birmingham B8 3TG, England, UK; ^2^ Department of Microbiology, Abia State University, Nigeria; ^3^ Department of Maternal and Child Health, School of Public Health, University of Alabama, Birmingham, AL, USA; ^4^ Division of Pediatrics and Community Health Ben Gurion University, Beer-Sheva, Israel

**Keywords:** insecticide-impregnated bednets, insecticides, malaria control, long-term exposure, health hazards, exposure, public health

## Abstract

The endemicity of malaria in tropical areas of the world persists, especially in countries south of Saharan Africa. The efforts and concerns invested by the World Health Organization and other health agencies to eradicate malaria are commendable. However, in spite of all these efforts, the loss in economic and human resources continues. In a previous report, the long-term health effects of insecticide-impregnated bednet (IIBN) use were highlighted with the expectation of attracting serious thoughts and further research on the issue. This present paper is an update on that expectation. Results from a comprehensive literature search show that not much work has been done on the effects of long-term exposure to IIBNs in combating malarial infection. The efficacy of IIBNs is not in question. What is in question is whether long-term exposure to IIBNs have any health effects. The aims and outcomes of the research found in the literature on the subject to date seem to support only the efficacy of the temporal use of plain bednets, but not the use of IIBNs, and do not tell much about the long-term effects of IIBN exposure. All pesticides are toxic by nature and present risks of adverse effects. While there is agreement that IIBNs can be effective in reducing malarial morbidity and mortality under field trials, a number of factors relating to their long-term-exposure health effects have yet to be determined. Further reliable research projects are recommended urgently. However, some of the anticipated behavioral effects caused by insecticidal use will be avoided by the use of untreated nets instead.

## INTRODUCTION

Malaria remains endemic in tropical and subtropical regions of the world. It still imposes a heavy burden on the health and economic productivity of populations in those regions (see Fig. 1). In the tropical and subtropical regions of the world, malaria causes 300–500 million clinical cases and about 2 million deaths each year. More than 90% of the deaths occur in sub-Saharan Africa and mostly among children under 5 years of age[1]. Malaria is a leading cause of perinatal mortality, low birth weight, and maternal anemia[2], while also having an economic drain both in terms of lost human productivity and health care costs. It is estimated that in sub-Saharan Africa, 15% of all disability-adjusted life-years (DALYs) is lost to malaria. It accounts for 40% of public health expenditure, 30–50% of inpatient admissions, and up to 50% of outpatient visits in areas with high transmission[1].

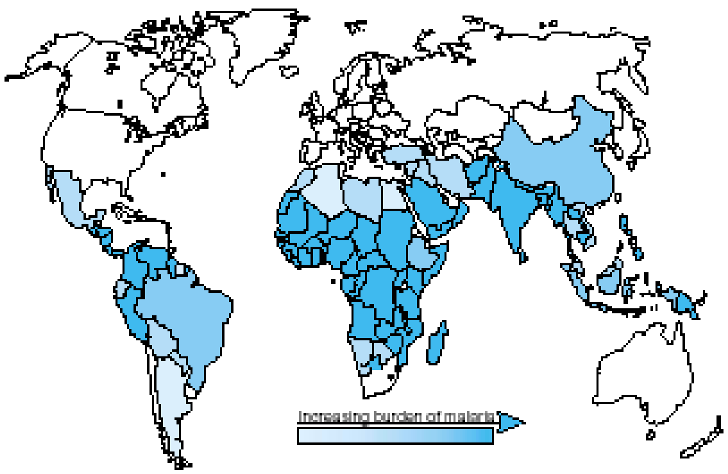

**FIGURE 1.** Malaria endemic regions. Source: Roll Back Malaria, 2002.

In addition, malaria impedes children's schooling and social development through absenteeism and permanent neurological damage associated with severe forms of the disease. The burden of malaria, combined with the present levels of HIV/AIDS and tuberculosis, presents a further serious challenge not only to public health in sub-Saharan Africa, but also to economic development. It has been estimated that malaria has slowed economic growth in African countries by 1.3%/year, which over 35 years amounts to a 32% reduction in the gross domestic product (GDP) of countries in the continent or an estimate at US$12 billion/year[1]. Over the last 2 decades, morbidity and mortality from malaria have been increasing due to deteriorating health systems, the impact of HIV/AIDS/TB, civil unrests, growing drug resistance, human migration, and population displacements.

## PROGRESS IN THE ROLL BACK MALARIA APPROACH

The World Health Organization (WHO), the United Nations Development Program (UNDP), the United Nations Children's Fund (UNICEF), and the World Bank founded Roll Back Malaria (RBM) in 1998, a global partnership for prevention and control of malaria. The goal was to halve the world's malarial burden by 2010 with the period 2001–2010 tagged as the *"United Nations Decade to Roll Back Malaria"* (http://www.who.int/inf-fs/en/InformationSheet02.pdf). The RBM partnerships include national governments, civil society and nongovernmental organizations, research institutions, international development agencies, and development banks. One key strategy that has emerged as a tool for malarial control under the leadership of RBM and its allies is the use of insecticide-treated mosquito nets

(ITMNs). As the WHO asserts, “there is no doubt about the effectiveness of ITMNs; the challenge is to scale up their use”[2]. This recommendation is based on field trials on the effectiveness of ITMNs[2,3], which demonstrated significant reductions in mortality following their use. In a recent report[4], Anyanwu et al. raised concerns about the long-term effects of insecticide-treated bednet (IIBN) exposures, especially on children and pregnant women. The present paper reviews the responses to the concerns raised previously, evaluates the extent of progress in the effectiveness of IIBN use, and assesses whether actions have been taken to minimize the inherent long-term health effects of IIBN exposure[4,5,6].

Jaffre[7] reported that most countries with endemic malaria have implemented national control programs under the RBM scheme. However, there has been practically no change in the burden caused by malaria in zones of highest transmission, especially in Africa. Therefore, five main areas in which social anthropology could make a positive contribution towards the implementation of the RBM program were suggested as follows[7]:

1. Improving mutual understanding between the population and heath care providers by describing popular systems for interpreting the disease.
2. Describing available care services and popular healing practices as a means of understanding the reasoning underlying access to care.
3. Analyzing the implications of recommended preventive measures for social behavior (e.g., “bed manners”) that must take into account the modalities of appropriation of the proposed innovations.
4. Improving the relationship between caregivers and patients by evaluating local moral perceptions.
5. Reducing the adverse effects of oververticalization of control programs by promoting opportunities for synthesis with projects initiated by community groups and local officials.

By undertaking work on these problem areas, it was anticipated that the preliminary and permanent pluridisciplinary teams could improve the current situation. However, recent reports [8] showed that the morbidity due to malaria was still very high (44.3% of cases) among children less than 5 years of age with 46.5% rate of hospitalization. The crude percentage mortality (129%) was also very high. Despite of this risk, percentage of pregnant women sleeping under an impregnated bed net and following chemoprophylaxis was 43.3% in the process of RBM in Benin. This percentage value seemed inauspicious for the unborn children [8]. There was therefore a consensus agreement for the use of non-impregnated bed nets instead. While it is accepted that use of IIBNs was only partly effective, other subjects expressed concerns stating that the use of IIBNs was tantamount to using as “a toxic family planning aid”[9]. Although the efficacy of insecticide-treated nets (ITNs) in malarial prevention is well documented, results show that malaria is perceived as a serious illness among pregnant women and children, and there is high awareness of the benefits of ITNs. However, few people, mainly because of their high cost and the perception that the chemicals used to treat them have dangerous effects on pregnancy and the fetus, use ITNs[10]. It is important to undertstand that malarial vectors and parasites have the genetic manipulative apparatus for constituting resistance to insecticides as well as antimalarial drugs after a short period of exposure and metabolic encounter. Now, pyrethroid-resistant mosquitoes are becoming increasingly common in parts of Africa. That is one of the reasons why Asidi et al.[11] suggested that it was important to identify alternative insecticides, which, if necessary, could be used to replace or supplement the pyrethroids for use on treated nets. However, any suggestion for the use of abandoned organophosphates is ridiculous and undesirable.

Preventing the death of children from malaria is worthy of international intervention and any measure to achieve this goal deserves unequivocal commendation. However, often with international health issues, resources and efforts are committed and abandoned because of unanticipated problems with sustainability[12], and limited operational research suggests that this may well be case with ITMNs. One area of the current initiative to promote the use of ITMNs that has received limited attention is operational research to examine the potential hazards and risks of long-term exposure to ITMNs. This is a serious omission given that products for impregnating mosquito nets with insecticides will be handled by untrained persons in uncontrolled settings in less-developed countries. In a recent report, Anyanwu et al.[13] emphasized that there was a propensity for hydrophobic organic compounds, such as those used in IIBNs, to cause adverse health effects. Suggestions were made concerning the importance of reliable scientific investigations into the long-term health effects of IIBNs. This paper is an attempt to contribute to the existing body of knowledge on this subject, with the hope that the ensuing argument will provide further impetus for debate and re-evaluation of the sustainability of ITMNs as a lead tool for malarial control in sub-Saharan Africa.

## INSECTICIDES IN THE CONTROL OF MALARIA

The word insecticide is used to describe any chemical that is applied against undesired insects that cause harm to animals, humans, and plants. All pesticides are toxic in nature. The risk of adverse effects from a pesticide depends on the toxicity of the chemical and the degree to which an organism is exposed. Health risks arising from the use of ITMNs primarily include exposure during storage and transport, during net treatment, and during use, exposure to vapors, or dermal or oral contact. There is limited literature on the health hazards associated with long-term exposure to ITMNs[14,15]. However, the neurotoxicity in humans of many insecticides has been studied and reviewed[16,17,18,19]. Evidence shows hat all organochlorines, organophosphates, carbamates, pyrethroids, and some fumigants used as insecticides are known neurotoxicants.

The use of chemical insecticides, such as organochlorines, to combat mosquitoes began in the 1940s. Over the years, several research findings have demonstrated that the use of chemical insecticides in controlling malarial vector had not only failed in its objective, but also induced toxic effects on humans and the environment[20]. It is unfortunate, therefore, that in less-developed countries, particularly those in Africa, insecticides such as DDT, Aldrin, and Diedrin are still being used without effective restriction[21]. Hence, the indoor and outdoor environmental dispersion of insecticides has become relatively ubiquitous as pollutants in human tissues, drinking water, soils, and food[22]. Their adverse toxic effects are mainly due to persistence in the environmental media over time and their consequent interaction with human biological systems, which may in turn lead to a plethora of neuropathological and behavioral health consequences[23].

## USE OF INSECTICIDES IN LESS-DEVELOPED COUNTRIES

In less-developed countries, particularly those in sub-Saharan Africa, pesticide user health and safety procedures are still almost nonexistent and minimally considered a serious issue in political and health discourse. During the rainy season, when the rate of malarial infection is high, people in the endemic areas use insecticides (including the “outlawed” DDT) to combat *Anopheles* mosquitoes indoors, with no awareness of the health risks. Their risk perceptions are very low, and self-protective behaviors are built on ignorance and limited health hazard awareness. People apply pesticides without protective masks and they usually spray or light the insecticide “coils” in their bedrooms before going to sleep[24,25].

In a situation such as this, it would be impossible to avoid direct pesticide inhalation and at least residual neurotoxic response to the chemical insecticide. To investigate the extent of the problem, the U.K. Pesticide Action Network (http://www.pan-uk.org/default.htm) undertook case study evaluations of the safe use of ITMNs in a number of African countries. In Gambia, for example, the group selected permethrin, the insecticide used to treat bednets in the country’s malarial control program[25]. Permethrin is imported into Gambia by the government and a number of aid agencies. There are 1300 villages involved in the program and studies have shown that the use of permethrin-impregnated bednets has reduced the number of fatalities from cerebral malaria. However, permethrin is a neuropoison and, as well as acting on the nervous system, can cause itching and burning sensations on exposed human skin. Field studies revealed significant risks of exposure at a number of stages of the chemical life cycle. These included the time when the pesticide is decanted from the large imported drums, when the bednets are impregnated by village health workers or by individuals in their own homes, when unlabelled containers are brought into the home, and from misuse of the chemical[25].

## Pyrethrins: Permethrin

Because of the adverse toxic effect of chemical insecticides, it became necessary to look for suitable compounds that would be efficacious, environmentally friendly, and nontoxic to humans. Unfortunately, “no such suitable alternative exists”[26]. Consequently, chemists resorted to such natural plant products as “alternative insecticides”; hence, pyrethrins obtained largely from the flowers of the *Chrysanthemum cinerariaefolium* plant were found to be effective[27]. The insecticidal activity of pyrethrin flowers has been known for over 2000 years, but the commercial production of pyrethrum began about 2 centuries ago[26,28]. However, it was only in 1920 that Cremlyn[28] demonstrated the chemical structure of the active insecticidal ingredient in the plant. Kenya is the world's largest producer of *C. cinerariaefolium*; other major producers are Russia and Japan. Each flower head contains 2–4 mg (c. 2%) of a mixture of active components that are extracted with light petroleum. It is from the chlorination of *C. cinerariaefolium* that permethrin used in IIBNs is derived[26,28].

## Pyrethrins Analogues

Several research activities were carried in the 1970s to synthesize analogues of pyrethrins, but it was Elliot and James[29] at the Rothamsted Research Station that made a breakthrough. Several compounds of pyrethrins were prepared including Allethrin, prepared from a racemic mixture of (R, S)-chrysanthemic acid, and bioresmethrin, in which the cyclopentenone ring of the natural product was replaced by the structurally simpler benzylfuran and yet the compound was extremely potent, although still short lived because of its photosensitivity[29]. The success of Elliot and James[29] led to the commercial development of permethrin[30,31]. Permethrin is supplied and marketed as an insecticide under several trade names, for example Ambush (ICI), Cooper powder (Wellcome Foundation Ltd), Permit (Pan Britannica Ltd), as Rentokil Musk control, and in mixtures with other substances[28].

## Permethrin-Impregnated Bednets

Field trials of permethrin-impregnated bednets against malarial vectors began about 2 decades ago and several follow-up studies were conducted and reported[32,33,34,35,36]. While results indicate reductions in mortality in the short term, little research has been conducted on the biomedical hazards associated with use of ITMNs and the long-term health effects of their use. Research attention has been meager, and data on acute and chronic health effects related to their toxic exposures are generally lacking[35], apart from a few recent reviews[13,37,38,39]. This suggests a need for a major research focus, with priority on long-term effects, particularly cancer, neurodevelopmental and neurobehavioral effects, long-term neurological dysfunction, and reproductive outcomes. No studies have been done or reported specifically on the clinical manifestations of ITMN neurotoxicity and neurobehavioral disorders at lower-exposure concentrations. Suitable populations at high risk should be studied including noncertified pesticide applicators, seasonal and permanent casual users, and their children. It is of interest to public health, especially, that the users of IIBNs be made aware that long-term exposure to an environmentally active neurotoxicant can be hazardous to health.

## NEUROTOXICITY AND NEUROBEHAVIORAL EFFECTS

All insecticides have both acute and chronic effects[40], and their use in agriculture and industry is largely regulated[41]. However, in spite of the known health risks associated with these, our review of reports on the benefits of ITMNs did not yield any information relating to the potential adverse effects of long-term exposure. A given public health intervention may have proven effectiveness in reducing morbidity and mortality, but only carefully planned studies examining negative impacts can reveal long-term adverse effects. The promotion of ITMNs as an important component of a malarial control program in malaria-endemic regions of the world should include studies on effects of long-term exposure. In the absence of such data, there would seem to be little justification for their continued use as a sustainable method for controlling malarial infection. The human central nervous system (CNS) and the peripheral nervous system (PNS) comprise highly specialized organs and tissues that are extremely sensitive to all chemical pollutants and can be injured or impaired by insecticide inhalation[42]. Certain features of neural tissues make them particularly susceptible to insecticide effects, namely, they are unable to replace lost and damaged cells; some of their components, such as myelin, have the tendency to accumulate and retain lipophilic substances, such as organic solvents; and they have a large surface area, which increases the likelihood of exposure to systematically distributed neurotoxicants[43]. Potentially serious neurotoxic effects of insecticides include peripheral neuropathy, extrapyramidal disorders, ataxia, sensory disorders, psychosis, CNS depression, and in extreme cases, death[44]. The physiological responses might not be manifest as overt disease effects, however, subclinical behavioral or neurological changes can be early indicators of an important disease process and can help to identify persons affected by exposures at stages that offer more effective opportunities for intervention[45].

Like other naturally occurring pesticides, all pyrethroids (including permethrin) are capable of affecting the heart rate of adult humans at exposures as little as 2 mg and can cause loss of memory[46]. Serious poisonings are frequent and in extreme cases may lead to death from respiratory failure[26,31,47]. Their toxic action takes place at the synapses, or junctions between nerve cells, where impulses are transmitted chemically through the acetylation of chlorine. When functioning normally, this reaction is rapidly reversed through the action of cholinesterase, so that stimulation ceases and the site is ready to receive a new impulse. The pyrethrins and other insecticides poison the nerves by inhibiting cholinesterase, which leads to the failure of nervous transmission, convulsions, and death[25,31,47]. Metabolically, their action on the nervous system may probably lead to hallucination, myoclonic phenomena, migrainous headaches, nausea, emesis, genetic mutation, and reproductive dysfunction.

### Carcinogenicity of Insecticide Exposures

The potential danger of human exposure to pesticides has become increasingly clear in the last 25 years and the association with cancer types[48,49] is now evident in several cancer registers in developed countries. In Africa, however, there are scarce (if any) cancer registers and records of cancer deaths are limited. Furthermore, cancer cases are very rarely reported as opposed to the developed countries, where strict regulations are in force. Probably, permethrin may cause damage to the immune system and lead to chromosomal abnormalities. It may also possibly modify the functions of liver enzymes and interfere with the conduction of nervous impulse.

Permethrin's cancer-causing capability probably depends on the length of exposure. This may occur when hydrogen atoms in the nucleotide bases undergo slight alterations in position. Hypothetically, for example, permethrin may act on the chlorine atoms of nucleic acids, thereby causing mutation. In addition, this nucleic complex may act as an immunosuppressant[50]. On the other hand, permethrin may displace adenine and if this replacement takes place during reproduction, mutation of the genes may occur. Note that the CG base pair (II) replaces the original AT (III, IV) base pair at the top of the DNA molecule, probably due to interference of permethrin. The DNA molecule replicates in the normal fashion, except that the permethrin that replaced the A mispairs with G, rather than with T, as A normally does. When this happens, the base is unable to pair with its normal complementary partner, but instead pairs with another base; for example, guanine may pair with thymine, or cytosine with adenine. If permethrin is incorporated in place of adenine, it may pair with guanine instead of thymine, causing a base substitution during cell division.

Normally, tautomeric shifts are only transitory states of a base and have little effect. If however, a tautomeric shift occurs at the time of DNA replication, the mispairing causes a change in the base sequence in the complementary chain that can be transmitted to future copies of the DNA — a substitution mutation. Tautomeric shifts occur because of natural chemical properties of the nucleotide bases and help to explain the normal spontaneous mutation rate. Like many other environmental agents, all insecticides also affect the rate of mutation. Additionally, variations in the mutation rate have been associated with biological factors such as age and sex[51]. Under these circumstances, it is possible that unidentified genetic diseases will occur in mating involving older fathers. The people who live in malaria-endemic regions of Africa are already environmentally exposed to insecticides and the additional exposure to ITMNs could enhance a tissue saturation point and increase the rate of genetic mutation since all chlorinated hydrocarbons are powerful mutagens[52].

## PUBLIC HEALTH PRIORITY ACTION

From the points raised so far, it is imperative that international health agencies and national governments re-evaluate the application of ITMNs as one of the strategies for combating malarial vectors, by instituting studies of long-term effects. One of the major problems reported in previous trials in Africa was the lack of awareness of pesticide-related health risks and hazards[53]. It is important that people are made aware that exposure to insecticides places them at increased risk of a variety of acute and chronic conditions including cancer[54,55]. Hazard characterization involving the re-examination of all available experimental animal and human data, and the associated doses, routes, timing, and duration of exposure, to determine qualitatively if and which brand of insecticide causes neurobehavioral and neurotoxic effects and under what conditions, must be undertaken.

It is possible that, from the hazard characterization and other relevant criteria, a health-related database can be generated and characterized as sufficient or insufficient for use in risk assessment. By combining hazard identification and some aspects of dose-response evaluation into hazard characterization, the evaluation and use of data for other purposes when quantitative information for setting reference doses (RfDs) and reference concentrations (RfCs) is not available will not be precluded[3,49,50,54,55]. Exposure assessment is important since it will assist in identifying human populations exposed or potentially exposed to insecticides, describe their composition and size, and present the types, magnitudes, frequencies, and duration of exposure to insecticide hazards. The exposure assessment would provide an estimate of human exposure levels for particular populations from all potential sources.

Since there have not been any scientific judgments made concerning the potential for IIBNs to cause health problems in humans, it is important that studies are carried out in this regard. Such research should adopt the National Research Council (NRC) recommendations, which define risk assessment as including some or all the following components: hazard identification, dose-response assessment, and exposure assessment and risk characterization[25,56,57]. Based on these recommendations, serious and urgent evaluation of IIBNs should be conducted through a research approach that is less fragmented and more holistic, more interactive, and that deals with recurring conceptual issues that cut across all stages of risk assessment. Such research should describe a more interactive approach by organizing the process around the qualitative evaluation of toxicity data (hazard characterization), the quantitative dose-response analysis, exposure assessment, and risk characterization. The hazard characterization should include deciding whether a pesticide has an effect by means of qualitative consideration of dose-response relationships, route, and duration of exposure. Efforts should be made to obtain information regarding personal characteristics, life style factors, and other variables that might be necessary for the risk assessment. Descriptive statistics of concentration levels should be computed to characterize the current extent of contamination. Time trends should be followed across the years, and homogeneous subgroups should be compared using multiple regression analysis to investigate associations between determining factors and specific insecticides. This approach would combine the information that is important when comparing the toxicity of ITMNs with potential human exposure scenarios. Many of the anticipated behavioral effects caused by insecticide use could be avoided by the use of untreated bed nets. This is supported by the evidence from studies on the efficacy of untreated bed nets to which untreated nets provided a reasonable degree of protection against malaria [4].The Present situation in the use of IIBNS
A recent study in the U.S. suggests that 80% of most people's exposure to pesticides occurs indoors and that measurable levels of up to a dozen pesticides have been found in the air inside homes. The amount of insecticides found in homes appears to be greater than can be explained by recent pesticide use in those households. Kakko et al.[54] report that the personal protective effect of pyrethroid-treated bednets against late-night biting mosquitoes is considerable, however, some biting of the occupants does occur, as shown by matching microsatellite alleles in mosquito blood meals to those of net occupants. They stated that when whole communities were provided with treated nets, ovarian age grading showed a reduction in mosquito survival rate and in the number of sporozoite-positive mosquitoes in malarious communities. Thus, a high percentage of coverage of all members of malaria-endemic communities is considered the most effective way of providing protection for highly malaria-vulnerable children and pregnant women. They also stated that there was evidence for reduced antimalaria antibody levels in children in communities where treated nets have long been used. However, overall benefits in reduced anemia and mortality are sustained. A high frequency of the kdr resistance gene has not prevented pyrethroid-treated nets from functioning, but it is important to develop alternative fabric treatments in case stronger forms of resistance emerge[57]. It must be emphasized that the efficacy of IIBNs is not in question. What is in question is whether long-term exposure to IIBNs has any health effects. However, because of possible interactions with immunity development, treated bednets may cause no effect at all, or even an increase in malaria morbidity and mortality in areas of high transmission. Curtis et al.[58] conducted a randomized controlled trial to assess the long-term effects of bednet protection during early infancy. A total of 3387 neonates from 41 villages in rural Burkina Faso were individually randomized to receive either bednet protection from birth (group A) or from age 6 months (group B). Primary outcomes were all-cause mortality in all study children and incidence of falciparum malaria in a representative subsample of the study population. They found that after a mean follow-up of 27 months, there were 129 deaths in group A and 128 deaths in group B rate ratio (RR) 1.0 (95% confidence interval [CI]: 0.78–1.27). They also found that falciparum malaria incidence was lower in group A than in group B during early (0–5 months) and late infancy (6–12 months) (RR 3.1, 95% CI: 2.0–4.9; RR 1.3, 95% CI: 1.1–1.6). They then concluded that their study provided additional evidence for the efficacy of ITNs in young children living in areas of intense malarial transmission.

One of the major problems we have in human health sciences research today is the problem of contradictory statistical “double speak”, which says little or nothing about the main issues at hand. For example, both the aim and outcome of the Curtis et al.[58] research seem to support only the efficacy of the temporal use of bednets, but not the use of IIBNs, and do not tell much about the long-term effects of IIBN exposures.

## CONCLUSION

Even in the interest of public health, the use of IIBNs without adequate risk assessment is unacceptable to a majority of people. Getting 60% of the world’s population at risk of malaria sleeping under IIBNs as targeted by RBM is a major operation that would involve unprecedented use of insecticides in less-developed countries. The negative effects of long-term exposure to these insecticides need to be balanced against the benefits from decreasing the burden of disease. In doing this, it has to be noted that in resource-poor settings, particularly Africa, what can be perceived as minimal risk may indeed become a major public health catastrophe when the risk combines with poverty, ignorance, illiteracy, and a chronic lack of resources for dealing with emergencies. Thus, a reassessment of the health risks associated with ITMNs is particularly important. In weighing the benefits of ITMNs against risks associated with their use, international development and health agencies should pay equal attention to other strategies that rely on environmental management and human behavior. Rather than conclude that the risks associated with long-term use of ITMNs are minimal compared with lives saved from malaria[14], a program of research should be instituted to identify the effects of long-term use on different segments of the population.

Procedures for monitoring exposure to ITMNs need to be instituted so that monitoring could be carried out on a regular basis, and to facilitate assessment of impacts and changes over time. Human behavior and the potential for unapproved misuse of insecticides should be addressed. Risk analyses should be based on actual levels of health and environmental exposure under the conditions of use. Training (including training of trainers) in insecticide use and management is important at all levels, and methods of disseminating information and knowledge about insecticides need to be instituted. Public awareness of potential risks associated with use and misuse of insecticides and of the health impact of insecticides is as essential in developing countries as it is in industrialized countries[3,25,39]. Given the potential risks associated with insecticide programs, promotion of ITMNs ought be subject to health impact assessment (HIA).
